# Can Procalcitonin Be an Accurate Diagnostic Marker for the Classification of Diabetic Foot Ulcers?

**DOI:** 10.5812/ijem.13376

**Published:** 2014-01-05

**Authors:** Nematollah Jonaidi Jafari, Mahdi Safaee Firouzabadi, Morteza Izadi, Mohammad Sadegh Safaee Firouzabadi, Amin Saburi

**Affiliations:** 1 Health Research Center, Baqiyatallah University of Medical Sciences, Tehran, IR Iran; 2 Department of Clinical Sciences, School of Veterinary Medicine, Ardakan University, Ardakan, IR Iran; 3 Chemical Injuries Research Center, Baqiyatallah University of Medical Sciences, Tehran, IR Iran; 4 Atherosclerosis and Coronary Artery Research Centre, Birjand University of Medical Sciences, Birjand, IR Iran

**Keywords:** Infected Diabetic Foot Ulcer, Procalcitonin, Inflammatory Marker, Diabetic Arteriopathy

## Abstract

**Background:**

The differentiation of infected diabetic foot ulcers (IDFU) from non infected diabetic foot ulcers (NIDFU) is a challenging issue for clinicians.

**Objectives:**

Recently, procalcitonin (PCT) was introduced as a remarkable inflammatory marker. We aimed to evaluate the accuracy of PCT in comparison to other inflammatory markers for distinguishing IDFU from NIDFU.

**Materials and Methods:**

We evaluated PCT serum level as a marker of bacterial infection in patients with diabetic foot ulcers. Sixty patients with diabetic foot ulcers were consecutively enrolled in the study. A total of 30 patients were clinically identified as IDFU by an expert clinician, taking as criteria for purulent discharges or at least two of manifestations of inflammation including warmth, redness, swelling and pain.

**Results:**

Procalcitonin, white blood cells (WBCs), erythrocyte sedimentation rate (ESR), and C-reactive protein (CRP), were found significantly higher in the IDFU group compared to the NIDFU group. The best cut-off value, sensitivity and specificity were 40.5 mm/h, 90% and 94% for ESR, 7.1 mg/dL, 80% and 74% for CRP, 0.21, 70% and 74% for PCT, and 7.7×10^9^/L, 66% and 67% for WBCs, respectively. The area under the receiver operating characteristic curve for ESR was the greatest (0.967; *P < 0.001), followed by CRP (0.871; P < 0.001), PCT (0.72*9; *P *< 0.001), and finally WBCs (0.721; *P* = 0.001).

**Conclusions:**

These results suggest that PCT can be a diagnostic marker in combination with other markers like ESR and CRP to distinguish infected from non-infected foot ulcers, when clinical manifestations are un specific. Additional research is needed before the routine usage of PCT to better define the role of PCT in IDFU.

## 1. Background

Diabetic foot ulcer (DFU) is one of the most common causes of mortality and morbidity, accounting for nearly two-thirds of all non-traumatic amputations perfumed in the US (1, 2). The lifetime risk of a DFU could be as high as 25% (3). Foot amputations may be preventable with prompt recognition and therapy (4). Patients with diabetes have an increased tendency to develop serious infections. Furthermore, after foot ulceration, the wound healing process may be prolonged, especially if the bacterial infection reaches deep tissues and bone. Fifty-nine percent of the diabetic foot amputations were attributed to infection (4). The successful treatment of an existing DFU is based on careful examination and classification of the wound. Although Some diabetic foot lesions are infected, others are not, and one of the most important prognostic factors for the outcome of DFU is infection. Also, infection is one of the International Working Group of the Diabetic Foot (IWGDF) classification items (5). The DFU infections may be present as superficial lesions, which sometimes may account for deep tissue involvement. 

Infection diagnosis in a DFU is not always simple and explicit. For the moment, most DFU infections are diagnosed clinically (on the basis of the presence of purulence or at least two cardinal manifestations of inflammation) or/and based on laboratory findings (6, 7). Because of the immune and host factor impairment, clinical symptoms may not be overt (8). Laboratory assessment should contain baseline and subsequent white blood cells (WBCs), inflammatory markers such as erythrocyte sedimentation rate (ESR), and C-reactive protein (CRP) which can be beneficial for monitoring response to treatment (9). Routine blood inflammatory markers (such as ESR and CRP) are not specific for bacterial infections and rise in almostany inflammatory process, while also accounting from important variation in relation to age, sex, and race. New effective, specific and sensitive markers of inflammation would help the quick detection of infection and prompt antibiotic therapy.

Recently, procalcitonin (PCT) has been introduced as an inflammatory marker and acute phase reactant, which may be especially useful in distinguishing bacterial infections (10-17). However, malaria, severe trauma, burning, and the medullary carcinoma of the thyroid can be causes of high PCT level in nonbacterial conditions (11). Few observational studies have recommended that PCT may be a reliable marker to distinguish bacterial infections in DFU (12, 14, 17). Limited data imply that increased PCT levels (> 0.5 ng/mL) have a greater diagnostic specificity than CRP in distinguishing bacterial infections (10, 15).

## 2. Objectives

In this study, the diagnostic accuracy of PCT was evaluated in comparison with other inflammatory markers as an indicator to make the distinction between infected and non-infected DFU, as the addition of antibiotics, costly medications, is justified only in the presence of bacterial infection.

## 3. Patients and Methods

### 3.1. Patients 

Sixty consecutive patients with diabetic foot ulcers hospitalized in the infectious diseases ward of Baqiyatallah Hospital, Baqiyatallah University of Medical Sciences, Tehran, IR Iran, between September 2009 and October 2010, were orderly registered. An extra group of healthy individuals (n = 30) was determined too. Patients with other infectious diseases like sepsis, urinary tract infection, pneumonia, meningitis, patients admitted due to surgery in the previous six weeks, with malignancy, with inflammatory diseases such as inflammatory bowel syndrome, rheumatoid arthritis or other rheumatologic disorders, patients receiving immunosuppressive treatment and with previous use of antibiotics were excluded from the study. The study had approval of Baqiyatallah University of Medical Sciences Ethics Committee, and all participants gave their informed consents.

Patients were assessed for diabetic foot infection by an infectious diseases expert, and a general physician was responsible for supervision on data collection. The guidelines of the Infectious Diseases Society of America (IDSA) and the IWGDF were used for clinical classification of IDFU by a specialist of infectious diseases. Localization (toe, metatarsal, mid foot/heel) and production of pus were recorded. Clinicians diagnosed infected diabetic foot ulcer (IDFU) according to the IDSA guidelines (9). Clinically, IDFU (or grade ≥ 2 of IWGDF) was identified by the presence of purulent discharges or at least two of the features of inflammation including warmth, redness, swelling or indurations, and pain or tenderness (9). Non-infected diabetic foot ulcer (NIDFU) in the IDSA classification was characterized as grade I of the IWGDF. At the first day of admission, before antibiotic therapy, blood samples were taken for the measurement of PCT, ESR, CRP, WBCs, fasting blood sugar (FBS) and glycated hemoglobin (HbA1c) levels. 

### 3.2. Laboratory Analysis

The blood taken for the analysis of PCT levels was centrifuged for 20 min after being protected at room temperature for 30 min. A BRAHMS PCT kit (BRAHMS Diagnostic, Berlin, Germany) was used for detection of PCT serum levels with a functional detection limit of 0.05 ng/mL. Levels of CRP, WBCs and ESR were assessed by the hospital biochemistry laboratory. The investigation was performed in a blind manner.

### 3.3. Statistical Analysis

Data were shown as mean ± standard deviation (SD). One-way ANOVA was used for the comparison among groups. T-test was used for the comparison of quantities. The receiver operating characteristic (ROC) curve has been drawn and the are aunder the ROC curve has been shown. With Youden’s J formulation (J = specificity+sensitivity-1), the best cut-off value was calculated. Specificity, sensitivity, and the negative and positive predictive values of the biochemical parameters were determined using the best cut-off value. A P value < 0.05 was considered as statistically significant. Statistical analysis was performed using SPSS software version 17 (SPSS Inc., Chicago, Illinois, USA).

## 4. Results

In this study, 30 patients with IDFU and 30 patients with NIDFU were enrolled. Patients treated with oral or intravenous (IV) antibiotic during the previous 6 months because of diabetic foot ulcers were excluded from the study. The demographic data of the three groups (healthy, IDFU and NIDFU) have been summarized in Table 1.

**Table 1.** Demographic Information of the Healthy, NIDFU and IDFU Groups a

Demographic	Healthy Group ^b^	NIDFU Group ^b^	IDFU Group ^b^	Total ^b^
**Number**	30	30	30	90
**Age, y**	38.1 ± 7.4	54.9 ± 8.6	61.4 ± 10.6	51.5 ± 13.2
**Diabetic age, y**	_	13.9 ± 5.6	15.1 ± 9.3	14.5 ± 7.6
**Sex (male/female)**	21/9	17/13	14/16	52/38
**FBS ^d^, mg/dL**	98.7 ± 17.5	128 ± 31.6^c^	215 ± 77.0^c^	147.2 ± 69.4
**HbA1c level, %**	5.6 ± 0.3	6 ± 1.3	9.3 ± 1.7^c^	7 ± 2

^a^ Infected diabetic foot ulcer was diagnosed clinically by the presence of suppurative discharge or at least two of the features of inflammation such as redness, warmth, swelling or induration and pain or tenderness (9).

^b^ Data are presented as mean ± SD.

^c^ P value < 0.05 is considered statistically significant.

^d^ Abbreviations: FBS, fasting blood sugar; HbA1c, glycated hemoglobin; IDFU, infected diabeticfoot ulcers; NIDFU, non-infected diabetic foot ulcer

With respect to age and gender, there was no statistically significant difference between the three groups (P > 0.05). Fasting blood sugar and HbA1c levels in the IDFU group were significantly higher than in the NIDFU group. Wound characteristics of the NIDFU and IDFU groups have been summarized in Table 2.

The levels of inflammatory markers have been shown in Table 3. The PCT levels in the IDFU group were significantly higher than NIDFU (P = 0.002) and control groups (P = 0.003). Also, the CRP levels in the IDFU group were significantly higher than the NIDFU (P = 0.001) and control groups (P = 0.001). The WBCs count in the IDFU group was significantly higher than the NIDFU (P = 0.001) and control groups (P = 0.001) and its level in the NIDFU group was significantly higher than the control group (P = 0.01). On the other hand, ESR in the IDFU group was significantly higher than the NIDFU (P = 0.001) and control groups (P = 0.001), and its level in the NIDFU group was significantly higher than the control group (P = 0.001).

**Table 2.** Wound Characteristics of IDFU and NIDFU Groups

Wound Localization	NIDFU ^a^ Group, No. (%)	IDFU Group, No. (%)
**Toe**	20 (66.7)	23 (76.6)
**Metatarsal**	4 (13.3)	3 (10.1)
**Mid Footand Heel**	6 (20)	4 (13.3)

^a^ Abbreviations: IDFU, infected diabetic foot ulcer; N: number of cases; NIDFU: non-infected diabetic foot ulcer.

**Table 3.** Inflammatory Markers in IDFU, NIDFU and Control Groups

Groups	PCT ^a^, ng/mL	CRP, mg/dL	WBCs, 109 /dL	ESR, mm/h
**Control (n = 30)**	0.10 ± 0.04	1 ± 1.40	6510 ± 1149	6.40 ± 6
**NIDFU (n = 30)**	0.33 ± 0.37	9.20 ± 5.30	8073 ± 2070 ^b^	29.10 ± 11.90 ^b^
**IDFU (n = 30)**	1.20 ± 2.10 ^b,c^	46.50 ± 46.50^b,c^	9846 ± 3662 ^b,c^	76.70± 30.10^b,c^

^a^ Abbreviations: CRP: C-reactive protein; ESR: erythrocyte sedimentation rate; IDFU:infected diabetic foot ulcer; n: number of cases; NIDFU: non-infected diabetic foot ulcer; PCT, procalcitonin; WBCs: white blood cells count.

^b^ P < 0.05:statistically significantfrom control group (healthy group).

^c^ P < 0.05:statistically significant from NIDFU group

Diabetic foot infection was diagnosed clinically by the presence of suppurative discharge or at least two of the features of inflammation such as redness, warmth, swelling or induration and pain or tenderness (9).

**Figure 1.**
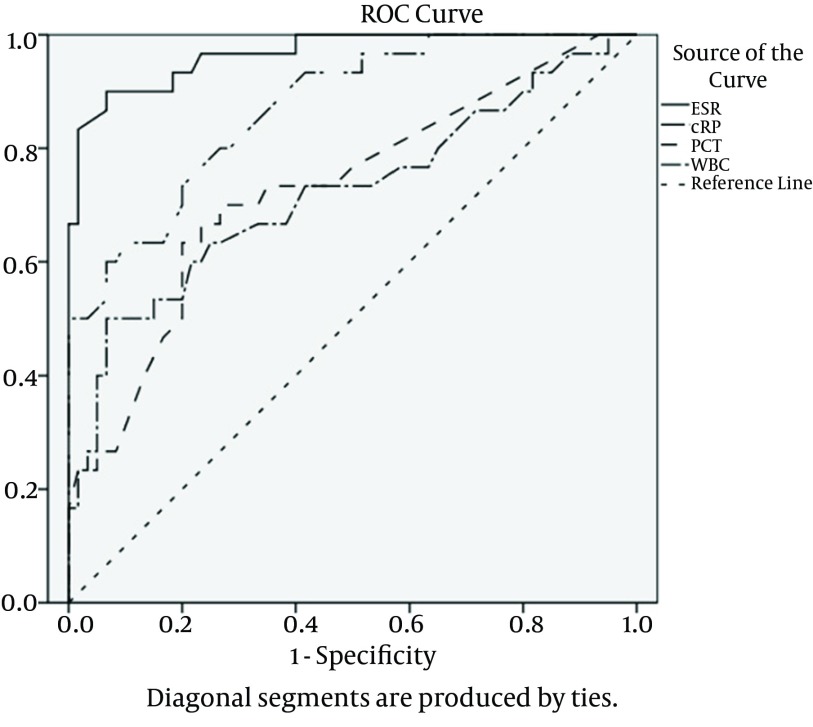
Receiver Operating Characteristic Curves of Inflammation Markers Abbreviations: ESR, erythrocyte sedimentation rate (solid line); CRP, C-reactive protein (doubled dotted and dashed line); PCT, procalcitonin (dashed line); WBC, white blood cell count (dashed and dotted line).

We calculated the area under the ROC curve to estimate the presence of bacterial infection in patients with diabetic foot ulcer (Figure 1). The area under the ROC curve for ESR was the greatest (0.967; P < 0.001), followed by CRP (0.871; P < 0.001), PCT (0.729; P < 0.001) and in the end, by WBCs (0.721; P = 0.001). The best cut-off value was 40.5 mm/h for ESR, 7.1 mg/dL for CRP, 0.21 ng/mL for PCT, and 7.7 x 10^9^/L for WBCs. Maximal sensitivity, specificity, and positive and negative predictive values have been displayed in Table 4 and the comparative diagram of their curves has been shown in Figure 1. 

**Table 4.** Specificity, Sensitivity, NPV and PPV of Inflammatory Markers for IDFU

Cut-off Value	Sensitivity, %	Specificity, %	PPV, %	NPV, %
**ESR^a^≥40.5 mm/h**	90	94	90	86
**ESR≥20 mm/h**	52	100	100	10
**CRP≥7.1 mg/dL**	80	74	80	46
**CRP≥5 mg/dL**	55	72	90	26
**PCT≥0.21 ng/mL**	70	74	70	50
**PCT≥0.5 ng/mL**	61	53	26	83
**WBC≥7.7×10^9^/L^a^**	66	67	66	40
**WBC≥10×10^9^/L**	80	60	40	90

^a^ Abbreviations: CRP, C-reactive protein; ESR, Erythrocyte sedimentation rate; IDFU, infected diabetic foot ulcer; NPV, negative predictive value; PCT, Procalcitonin; PPV, positive predictive value; WBC, white blood cells count.

The area under the curve was 0.967 for ESR, 0.871 for CRP, 0.729 for PCT and 0.721 for WBCs.

## 5. Discussion

The DFU is one of the most important complications of diabetes, which has a long and difficult healing process. These wounds can become infected and progress and could cause osteomyelitis and sepsis. Therefore, prevention and treatment of infected DFUs with antibiotics are very important. Different methods have been proposed for distinguishing between IDFU and NIDFU, but clinical findings are the most valid criterion yet. However, this method relies on individual expertise (9). Considering the possible errors in results of clinical assessment or laboratory tests, it erroneous to provide the appropriate treatment protocol in accordance with a single laboratory report. Occasionally, suspicious consequences, such as failure of an ulcer could prompt for infection to be cured within the expected time (18). Recently, PCT has been suggested as an important marker of inflammation, which increases in inflammatory processes, especially bacterial infections (localized or bacteremia). The PCT values have a progressive increasing pattern in bacterial infections, while the elevation is only mild in other inflammatory conditions (11, 19-21). Although PCT sensitivity and specificity are considered to be less strong than ESR or CRP to indicate IDFU, this study showed that PCT, like other inflammatory markers, can prove helpful in diagnosing the infection. The present study demonstrated that ESR is the most sensitive and specific inflammatory marker distinguishing IDFU from NIDFU. Although Several studies have been performed to determine the predicting and distinguishing role of PCT in various infections, only two similar studies have surveyed the role of PCT in distinguishing infected diabetic foot wounds from non-infected ones (14, 17).

In the study of Uzun et al., ESR, WBC and PCT had a decisive role in identifying diabetic foot wound infection, but CRP did not have a significant role, a finding inconsistent with the results of the present study (17). Also, the results reveal that PCT, among all the inflammatory markers, have the highest area under the curve and the greatest statistical significance in relation with infection. Although Seven of the 27 with an identified IDFU were also diagnosed as having osteomyelitis (by the probe to bone test),in our research, these were not analyzed separately (17).

Jeandrot et al. reported that PCT sensitivity and specificity, compared to other inflammatory markers (orosomucoid, haptoglobin, albumin, CRP, WBC, and neutrophils count) are not superior in distinguishing infected from non-infected diabetic foot wounds. In the aforementioned investigation, CRP was the most useful marker, having the highest sensitivity and specificity according to the DFU classification. Although, In the study of Jeandrot et al. CRP was introduced as the most sensitive and specific marker, in our study, specificity and sensitivity of CRP were, on one hand, less significant than ESR and, on the other hand, more than PCT or WBC (14).

The higher efficiency of ESR in denoting infection, compared with PCT, could be rationalized by the mild nature of infection in low grade diabetic foot wounds. Our study results confirmed that a higher level of PCT is presents in higher grades of IDFU. PCT level is usually higher in patients with severe and systemic infection (22).

Sensitivity is more important in differentiating these patients, and the highest sensitivity was obtained when the two markers (such as CRP and PCT, or ESR and PCT) were considered together, a finding previously reported in both the studies of Jeandrot et al. and Uzun et al. (14, 17). Since patients needed to be antibiotic free for at least six months, the sample sizes were small in the three studies, considering patients with history of DFU.

The normal level of PCT is very low (< 0.5 ng/mL). In bacterial infections, the amount of PCT may be observed to reach values a hundred times higher (23). In the present study the best cut-off value for IDFU diagnosis was 0.21 ng/mL for PCT (sensitivity, 70%; specificity; 74%, PPV, 70%; NPV, 50%).

Procalcitonin levels, before the study of Uzun et al. (17) and Jeandrot et al. (14), had been shown to increase remarkably only during severe bacterial infections with systemic manifestations. However, IDFU does not always manifest with such an obvious clinical picture (18, 23). Moreover, it should be supposed that it has not been regarded as a helpful marker, when used alone, because it does not increase markedly in local infections. In most clinical laboratories, measuring PCT serum level is not possible easily. Considering that the PCT level is higher in higher grade diabetic foot wounds and it is more effective than other laboratory markers in diagnosing bone infection (13), it can be used in the differentiation of bone involvement in diabetic foot ulcers (12). Finally, although PCT is a promising inflammatory marker, it seems that it is not more effective and useful than other classic markers (such as ESR or CRP) for classifying the infected diabetic foot ulcer from non-infected ones. Procalcitonin is not a specific marker for inflammation in some patients (such as diabetic patients with DFUs) yet. it is important to know whether there is a considerable inflammatory process or not.

There were some limitations in this study, which hinder a definite conclusion. In most hospital laboratories, PCT analysis is not routinely available. Also, controversy exists about the reliability of PCT level in the aforementioned studies because of the variability in outcome by age, pathogen and type of infection (11, 16, 19-22). There is a considerable difference in age and gender ratio between the healthy group and patients with diabetic foot, and this difference can interfere with our conclusion and it was one of our limitations. Further investigations are required to better clarify the usefulness of PCT for distinguishing IDFU from NIDFU.
